# Ecosystem Services and Their Driving Forces in the Middle Reaches of the Yangtze River Urban Agglomerations, China

**DOI:** 10.3390/ijerph17103717

**Published:** 2020-05-25

**Authors:** Wanxu Chen, Guangqing Chi, Jiangfeng Li

**Affiliations:** 1 Department of Geography, School of Geography and Information Engineering, China University of Geosciences, No. 388 Lumo Road, Wuhan 430074, China; cugcwx@cug.edu.cn; 2 Department of Agricultural Economics, Sociology, and Education, Population Research Institute, and Social Science Research Institute, The Pennsylvania State University, 112E Armsby, University Park, PA 16802, USA; 3 Department of Land Resource Management, School of Public Administration, China University of Geosciences, Wuhan 430074, China

**Keywords:** land-use/land-cover change, ecosystem services, driving forces, spatial regression, Middle Reaches of the Yangtze River Urban Agglomerations, China

## Abstract

The impact of human activities on ecosystems can be measured by ecosystem services. The study of ecosystem services is an essential part of coupled human and natural systems. However, there is limited understanding about the driving forces of ecosystem services, especially from a spatial perspective. This study attempts to fill the gap by examining the driving forces of ecosystem services with an integrated spatial approach. The results indicate that more than US$430 billion of ecosystem services value (ESV) is produced annually in the Middle Reaches of the Yangtze River Urban Agglomerations (MRYRUA), with forestland providing the largest proportion of total ESV (≥75%) and hydrological regulation function accounting for the largest proportion of total ESV (≥15%). The average ESV in the surrounding areas is obviously higher than those in the metropolitan areas, in the plains areas, and along major traffic routes. Spatial dependence and spatial spillover effects were observed in the ecosystem services in the MRYRUA. Spatial regression results indicate that road density, proportion of developed land, and river density are negatively associated with ecosystem services, while distance to a socioeconomic center, proportion of forestland land, elevation, and precipitation are positively associated with ecosystem services. The findings in this study suggest that these driving factors and the spillover effect should be taken into consideration in ecosystem protection and land-use policymaking in urban agglomerations.

## 1. Introduction

Urban agglomerations in China have become the main spatial carrier of urbanization [1]. Human activities in urban agglomerations have increasingly intensified land-use/land-cover change (LULCC) and exacerbated the evolution of ecosystem services [2,3]. Clearly identifying the evolutionary mechanisms of ecosystem services in urban agglomerations carries great significance for policy implications in land-use planning and ecosystem management. However, there is limited understanding about the determinants of ecosystem services from a spatial perspective. Within this context, it is crucial to characterize the spatial pattern of ecosystem services and explore their driving forces in urban agglomerations.

More specifically, an accurate assessment of ecosystem services is the foundation for studying the evolutionary mechanisms of ecosystem services, yet the assessment of ecosystem services and the study of the evolutionary mechanisms of ecosystem services in the emerging urban agglomerations in China remains incomplete. The Middle Reaches of the Yangtze River Urban Agglomerations (MRYRUA), an emerging urban agglomeration in China composed of the Wuhan metropolis, the Changsha–Zhuzhou–Xiangtan metropolis, and the Poyang Lake city group, was selected as the study area to explore land-use features and identify the driving forces of ecosystem services with spatial regression models. Geographically, the MRYRUA is located at the intersection of the east–west and north–south development axes of China, and it is an important economic center. The Yangtze River Middle Reach City Group Development Plan, implemented in 2015, proposed a strategic positioning for building a new growth pole for China’s economic development, a new type of urbanization leading zone in central and western China, an inland open cooperation demonstration zone, and a “two-type” (resource-saving and environmentally friendly society) social construction leading zone in China [4]. Thus, it is beneficial to examine the evolutionary mechanism of ecosystem services in this fast-developing urban agglomeration. To that end, this study has three specific research aims: To explore the spatiotemporal features of LULCC and the spatial pattern of ecosystem services in the MRYRUA from 1995 to 2015; to examine the driving forces of ecosystem services based on spatial aspects; and then to explain related policy implications based on the spillover effect found in this study.

The term “Ecosystem Services” was first proposed by Ehrlich and Ehrlich (1981) based on the concept of “Environmental Services” proposed by Carroll and Wilson (1970) and the concept of “Natural Services” proposed by Westman (1977) [5,6,7]. Although there is not a uniform definition of ecosystem services, the general consensus is that ecosystem services are the benefits obtained by human beings from ecosystems; that the natural ecosystems and the resources needed for human survival and development ultimately come from natural ecosystems, the subject of ecosystem services; and that ecosystem services are reflected by the conditions and processes of the ecosystem itself [6,7]. The increasing demand for ecosystem services but the scarcity of ecosystem services has brought up increasing attention to define and estimate the value of ecosystem services. One way is to monetize these tangible or intangible natural capitals, which can reflect the value brought by ecosystem services and provide the basis reference for management decisions.

The ecosystem services classification and assessment framework proposed by Costanza et al. (1997) has pushed ecosystem services research into a new phase [8]. An increasing number of comprehensive ecosystem services studies concerning ecosystem services assessment, evolutionary mechanism, trade-offs and synergies, flows, and budgets have previously been carried out [9,10,11,12]. However, there is still work to be done in the study of ecosystem services [13]. For example, the existing literature about the driving forces of ecosystem services usually ignores the spatial dependence and spatial spillover effects. However, ecosystem services in one unit are influenced by individual elements as well as by the provision capacity of ecosystem services in neighboring spatial units; that is, ecosystem services in one unit are likely to deteriorate if the ecosystem services in surrounding units deteriorate [14]. Considering only individual elements may miss or distort the truth of the evolutionary mechanism of ecosystem services. To date, the binary logistic regression model [15], gray correlation analysis [16], and ordination analysis [17] have been widely used to examine the driving forces of ecosystem services, while only a few studies identified the spatial determinant of ecosystem services with spatial regression models [9,10]. In an increasingly connected world, the spatial interaction features of ecosystem services and spatial determinants provide important information about the coordinated management of cross-regional land use and ecological protection in urban agglomerations. Thus, the spatial autocorrelations and spatial spillover effects should be taken into consideration in understanding the driving factors of ecosystem services.

The evolution of regional ecosystem services takes place under the combined influences of physical, human, and neighborhood factors, as well as land-use policy and planning [16,17,18,19]. Physical elements, such as elevation, hydrological conditions, and climate, have dominant influences on the spatial distribution pattern of ecosystem services [20,21]. The provision capacity of ecosystem services varies substantially at different elevations by different hydrological conditions and climate [22,23]. However, we live on an increasingly human-dominated planet, and the impact of human activities on the terrestrial ecosystem has dramatically exceeded those of the physical elements [24]. The human factors affecting the evolution of ecosystem services are both direct and indirect. The chief direct factors are land-use change [25], afforestation [26], deforestation [27], and land reclamation [28], among others. The primary indirect factors include urbanization [29,30], economic growth [16], population migration [31,32], traffic accessibility [33], and land-use policies [34].

Urbanization is a complex process, as are economic agglomeration, population agglomeration, and the increased demand for developed land and ecological land in urban areas [35]. Urbanization-led LULCC has profoundly transformed ecosystem services [3] and the proportion of developed land should be an essential consideration in the evolution of ecosystem services. High population density and intense economic activities in urban areas have resulted in large-scale modification of ecosystem services [36]. The construction of infrastructure, such as highways and railways, promotes economic growth and population migration [37], thereby affecting LULCC and the provisioning capacity of ecosystem services. Proximity factors, such as distance to city centers, are closely associated with other socioeconomic factors that should also be considered [38].

## 2. Materials and Methods

### 2.1. Study Area and Data

**Figure 1:**
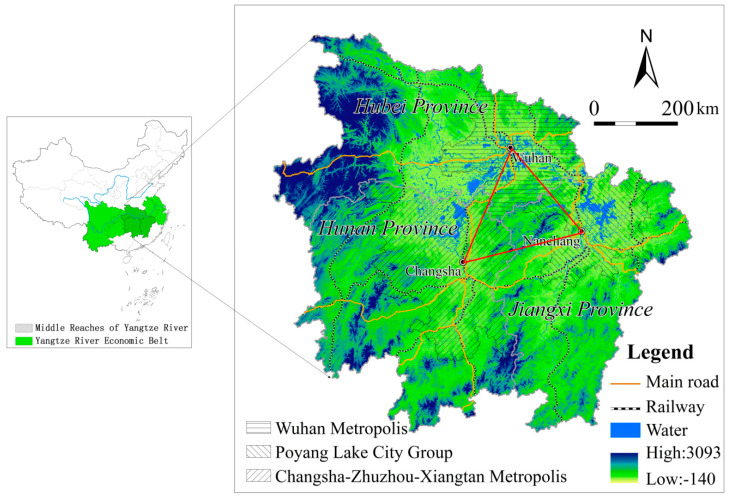
Location of the study area in China.

The MRYRUA in this study consists of three provinces: Hunan, Hubei, and Jiangxi (Figure 1). The MRYRUA is located in the transitional zone between China’s second and third steps. The types of landforms in the study area are complex and diverse, with significant spatial differences. The overall terrain is high in the west and low in the north. There are many types of landforms, including plains, hills, and mountains in the MRYRUA. The MRYRUA is surrounded by the Wu Mountains and Xuefeng Mountains in the west, Nanling Mountains in the south, and Wuyi Mountains in the east. The area is rich in farmland, forestland, water resources, and biodiversity, all of which play an important role in grain-producing and ecosystem services provisioning in China. With the Wuhan megalopolis, Changsha–Zhuzou–Xiangtan urban agglomerations, and Poyang Lake city group as its development centers, the MRYRUA has become an emerging national-level urban agglomeration in China. Rapid development in the MRYRUA greatly promoted the land-use transition and the deterioration of ecosystem services. During the period of 1995–2015, deforestation, afforestation, land reclamation, and returning sloping farmland to forestland occurred frequently in the MRYRUA, causing a dramatic effect on regional ecosystem services. In the context of the important strategic position of the MRYRUA and the environmental problems, it is necessary to study the supply capacity of the ecosystem services and their driving mechanism.

The 30-m resolution LULCC dataset was downloaded from the Data Center for Resources and Environmental Sciences, Chinese Academy of Sciences (RESDC) (http://www.resdc.cn) [39]. Landsat TM/ETM+ remote sensing imagery was the primary source of data for the LULCC dataset. Using the human–computer interactive interpretation method, Liu et al. constructed the national LULCC dataset in China at 5-year intervals [39]. According to specific research needs, the land uses are divided into six first-level types—farmland, forestland, grassland, water area, construction land, and unused land. To ensure the quality of the LULCC datasets, nationwide field surveys were conducted; the overall accuracy was over 90% [39,40,41].

### 2.2. Dependent Variables

The theoretical framework for the measurement of ecosystem services value (ESV) proposed by Costanza et al. (1997) has greatly promoted the study of the assessment of ESV globally [8]. Xie et al. (2003, 2008) revised the ecosystem services classification and equivalent table based on the expertise of ≥700 ecologists in China using the method of Costanza et al. (1997) [42,43]. Specifically, ecosystem services were reclassified into four main types and nine subtypes. The equivalent table of ESV was modified with the concept of equivalent value per unit area. The economic value of grain production per unit area of farmland was assumed to be 1. The equivalent value per unit area of other ecosystem services was identified based on their relative importance to grain production of farmland. The ESV equivalent factor per unit area was defined as equal to 1/7 of the average economic value of grain production per unit area of farmland. Chen et al. (2019a) calculated the ESV equivalent value ($US344.927/(hm^2^ a)) based on the grain yield data and the grain price data in the MRYRUA [44] (Equation (1)). Ecosystem services have been localized based on biomass, but biomass was not completely positively correlated, particularly in the water areas and wetlands [42]. However, the water area and wetlands in the MRYRUA are vast, contributing more than 10% of the total ESV [44] Chen et al. (2019b) further revised the ESV equivalent value based on the biomass of farmland (Equation (2)). [45] Based on the results of Chen et al. (2019b) [45], we took the average ESV of each county as the dependent variable in 1995, 2005, and 2015. The equations are as follows:

(1) $$EV=\frac{1}{7}\times \sum \frac{h_{r}\times p_{r}\times q_{r}}{M}$$

(2) $$ESV_{corrected}=\frac{VCI_{k}}{\bar{VCI}}\times \sum_{j=1}^{m}\sum_{i=1}^{n}\left(LUC_{i}\times EV_{ij}\right)$$

where *EV* is the economic value of grain production per unit area of farmland (ESV equivalent value; dollars/(hm^2^· a)); *h_r_* is the sown area of the *r*th grain crop (hm^2^); *p_r_* is the average price of the *r*th grain crop ($/ton); *q_r_* is the per unit area yield of the *r*th grain crop (ton/hm^2^); *M* is the sown area of all the grain crops (hm^2^); and *ESV_corrected_* indicates the corrected ESV. 

*VCI* indicates the average vegetation condition index computed from the normalized difference vegetation index of the farmland; $\bar{VCI}$ indicates the annual average *VCI*; *VCI_k_* is the average *VCI* of the *k*th county; *EV_ij_* is the *j*th category of ESV equivalent value for the *i*th land use type; and *LUC_i_* represents the area of the *i*th land-use type.

#### 2.2.1. LULCC in the MRYRUA from 1995 to 2015

Forestland in the MRYRUA accounted for the largest proportion of the total coverage (more than 58%), followed by farmland (30%). Land use in the MRYRUA changed significantly during 1995–2015. Forestland increased slightly from 1995–2005 (0.19%), while a significant decline was witnessed during 2005–2015 (0.60%). The area of farmland showed a continuous downward trend (−0.24% during 1995–2005 and −0.79% during 2005–2015). However, a continuous increase can be found in construction land (0.09% during 1995–2005 and 1.12% during 2005–2015) and water areas (0.26% during 1995–2005 and 0.21% during 2005–2015) (Table 1). Several important trends and transitions between different land-use types are also worth noting during 1995–2015. The most significant transitions happen between farmland and forestland: 5906.48 km^2^ of forestland converted to farmland, and 5459.57 km^2^ of farmland converted to forestland. For farmland and construction land, 5001.00 km^2^ of farmland were converted to construction land, while only 781.48 km^2^ of construction land were converted to farmland (Table 2). A significant imbalance can be found in the amount of cultivated land transferred in and transferred out during 1995–2005 and 2005–2015. The possible reason is that rapid urbanization took over a large amount of farmland, resulting in a rapid decline in the proportion of farmland. Additionally, since the implementation of the Farmland Balance Policy, the same amount of farmland should be reclaimed to make up for the farmland occupied by construction land, resulting in a large amount of forestland being converted to farmland. At the same time, due to the policy of returning farmland to forestland and the implementation of a series of ecological engineering projects, a large amount of farmland was converted into forestland during the study period.

#### 2.2.2. ESV in the MRYRUA from 1995 to 2015

The average ESVs in the MRYRUA in 1995, 2005, and 2015 were US$7724.03/hm^2^ · a, US$7817.47/hm^2^ · a, and US$7877.02/hm^2^ · a, respectively, documenting a gradually increasing trend during the period studied. Forestland provided more than 75% of the ESV in the MRYRUA, while farmland and water areas provided more than 10% of the total ESV (Table 3). The other land-use types, including grassland and unused land, provided only a small proportion of ESV. Among all the ecosystem functions, the hydrological regulation function accounted for the largest proportion of the total ESV (15.79% in 1995, 15.91% in 2005, and 15.98% in 2015). During 1995–2005, the 9 categories of ecosystem functions provided by grassland and unused land decreased, while the ecosystem functions provided by farmland, forestland, and water areas increased. The hydrological regulation function provided by water areas increased the most (US$1101.73 million), while the soil formation and retention function provided by grassland decreased the most (−US$122.97 million). During 2005–2015, only the ecosystem functions provided by farmland decreased. The soil formation and retention function provided by farmland decreased the most significantly (−US$228.37 million). The hydrological regulation function provided by grassland increased the most (US$528.70) during 2005–2015. 

**Figure 2:**
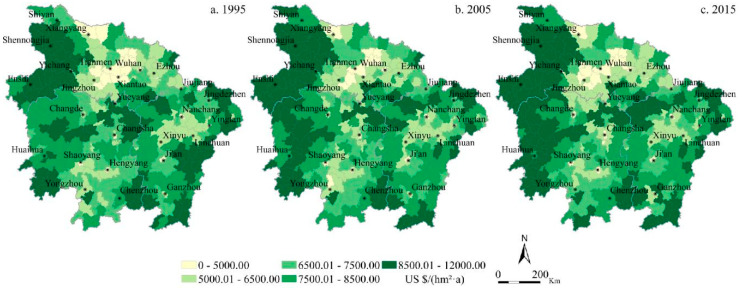
The spatial pattern of ecosystem services supply capacity in the MRYRUA from 1995 to 2015. (**a**) Ecosystem services supply capacity in 1995. (**b**) Ecosystem services supply capacity in 2005. (**c**) Ecosystem services supply capacity in 2015.

In terms of spatial distribution, the spatial pattern of the average ESV in the MRYRUA is relatively stable, and the low-value areas of the average ESV are distributed primarily in the Jianghan Plain, the Poyang Lake Plain, and the Dongting Lake Plain, especially in the key cities and surrounding areas (Figure 2). The high-value areas of the average ESV are mainly south of the Dabie Mountains and Wushan in the western area of Hubei, the Xuefeng Mountains in the central and western part of Hunan Province and Nanling in the southern part of Hunan Province, the Wuyi Mountains in the eastern region of Jiangxi Province, and the Luoxiao Mountains between Jiangxi Province and Hunan Province. The supply capacity of ESV provided by ecosystems varied significantly due to the significant differences in natural conditions and socioeconomic development levels in mountainous and plain areas.

### 2.3. Independent Variables

Based on the driving factors found in previous studies that impact ecosystem services, for this study we selected population density, road density, and proximity to represent human factors, and selected elevation, slope, temperature, precipitation, and river density to represent physical factors [16,17,18,46]. The proportion of forestland and the proportion of developed land were chosen to represent the land-use pressure [28]. Because a large number of variables may lead to a multicollinearity problem, a variance inflation factor (VIF) test was performed on the independent variables. After the VIF test, a set of factors was selected as independent variables (Table 4).

### 2.4. Regression Analysis

#### 2.4.1. Spatial Correlation Analysis

The spatial interaction and spatial diffusion assume that the attributes at one location are interdependent with those at other locations [47]. Global spatial autocorrelation and local spatial autocorrelation are commonly used to measure their spatial relationships. The regional spatial dependence of the average ESV was measured with the global Moran’s *I* index and local Moran’s *I* Index (LISA). Specifically, global spatial autocorrelation was used to verify the overall aggregation of the specific phenomena or attribute values in the spatial distribution of the entire region, while the local Moran’s *I* was used to measure the degree of spatial difference between a certain area and its surrounding areas. The scatter plot of Moran’s *I* provided a clear spatial clustering pattern of average ESV. The first and third quadrants in the scatter plot were, respectively, the high–high and low–low types; and the second and fourth quadrants were, respectively, the low–high and high–low types. The LISA map helps in understanding whether any of the spatial patterns are significant. Based on the statistical hypothesis testing, the Z_score_ ≥ 1.96 or Z_score_ ≤ –1.96 showed that the spatial autocorrelation was statistically significant at the 5% level. GeoDa095i software (University of Chicago, Chicago, IL, USA) was employed in this study to test the spatial autocorrelation of the county-level average ESV in the MRYRUA in the years 1995, 2005, and 2015 using the queen’s contiguity weight method [48]. The equations are as follows:

(3) $$I=\frac{n}{\sum_{i=1}^{n}\sum_{j=1}^{n}W_{ij}}\times \frac{\sum_{i=1}^{n}\sum_{j=1}^{n}W_{ij}\left(AESV_{i}-\bar{AESV}\right)\left(AESV_{j}-\bar{AESV}\right)}{\sum_{i=1}^{n}{\left(AESV_{i}-\bar{AESV}\right)}^{2}}$$

(4) $$Z(I)=\frac{I-E\left(I\right)}{\sqrt{Var(I)}}$$

(5) $$I_{i}=\frac{n\left(AESV_{i}-\bar{AESV}\right)\sum_{j=1}^{n}W_{ij}\left(AESV_{j}-\bar{AESV}\right)}{\sum_{i=1}^{n}{\left(AESV_{i}-\bar{AESV}\right)}^{2}}$$

where *AESV_i_*, *AESV_j_* are the spatial observations in position *i* and *j*, respectively; $\bar{AESV}$ is the average value of ecosystem services; and *W_ij_* is the spatial weight connection matrix (*i*, *j* = 1,2,3 ... *n*). *E(I)* and *Var(I)* are, respectively, the expected value and the variance of Moran’s *I*. The global Moran’s *I* value generally ranged from –1 to 1. Moran’s *I* values > 0 indicate positive spatial autocorrelation, Moran’s *I* values < 0 indicate negative spatial autocorrelation, and Moran’s *I* approaching 0 indicates a random distribution pattern.

#### 2.4.2. Spatial Regression Analysis

The spatial effects of the independent variables have often been neglected in previous research about ecosystem services, and it is commonly assumed that the effects are spatially independent and identically distributed. Using an ordinary least squares (OLS) model (Equation (4)) may lead to an incomplete explanation of the regression results because it ignores the potential spatial effects [49,50]. In this study, we use three spatial regression models, including the spatial lag model (SLM) (Equation (5)), spatial error model (SEM) (Equation (6)), and spatial error model with lag dependence (SEMLD) (Equation (7)) [37,50]. The SLM assumes spatial autocorrelation occurs in the dependent variable, emphasizes the neighborhood effect, and considers the phenomenon of spatial diffusion (the spillover effect) in the dependent variable across geographic units [51]. The SEM focuses on the neglected and unobserved spatial interdependencies among variables. The SEMLD is a spatial autoregressive model augmented by adding the spatially lagged dependent variable to the SEM. All the models were conducted in GeoDa095i at the county level. We identified the performance of the four models based on the log-likelihood value, the Akaike information criterion (AIC), and the Schwarz criterion (SC). The equations are given in Equations (6) through (9):

(6) $$AESV_{t}=X_{t}\beta +\varepsilon$$

(7) $$AESV_{t}=X_{t}\beta +\rho W_{1}AESV_{t}+\varepsilon$$

(8) $$AESV_{t}=X_{t}\beta +\varepsilon ,\varepsilon =\lambda W_{2}\varepsilon +\xi$$

(9) $$AESV_{t}=X_{t}\beta +\rho W_{1}AESV_{t}+\varepsilon ,\varepsilon =\lambda W_{2}\varepsilon +\xi$$

where *AESV_t_* is the matrix of dependent variable in time *t*; *X_t_* is an *n* × *k* independent variables matrix in year *t*; *n* is the number of study units; *k* is the number of independent variables; *β* is a vector of coefficients of *X_t_* indicating the influence level of the independent variables on the dependent variables; *ρ* is a spatial lag parameter; *ε* is a random error term vector; *λ* is a spatial error parameter; and *W*_1_ and *W*_2_ are spatial weight matrices for the lag term and the error term, respectively.

## 3. Results

### 3.1. Spatial Autocorrelation

**Figure 3:**
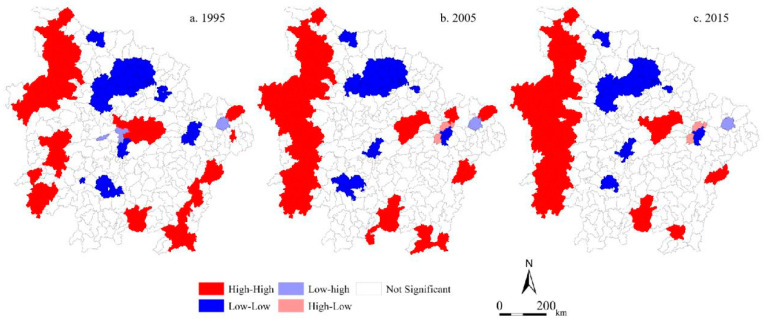
LISA clustering of ecosystem services supply capacity in the MRYRUA from 1995 to 2015. (**a**) LISA clustering of ecosystem services supply capacity in 1995. (**b**) LISA clustering of ecosystem services supply capacity in 2005. (**c**) LISA clustering of ecosystem services supply capacity in 2015.

The exploratory spatial data analysis method was used to further study the spatial distribution of aggregation and abnormalities of average ESV and the relationship of average ESV with the adjacent areas in the MRYRUA. Geoda950i software was used to calculate the Moran’s *I* of the average ESV from 1995 to 2015 in the MRYRUA. The global Moran’s *I* analysis of the average ESV indicated that the positive global Moran’s *I* of the average ESV (0.539 in 1995, 0.576 in 2005, and 0.591 in 2015) in the MRYRUA showed that counties with a high or low average ESV in the MRYRUA exhibited a significant agglomeration distribution pattern. Moreover, the gradual increasing trend of global Moran’s *I* from 1995 to 2015 indicated that the agglomeration tendency in the MRYRUA was strengthened. Additionally, the observed values of global Moran’s *I* and expected values (E(I)) did not change significantly, and *p* was significant at 0.01%, indicating that the average ESV in the MRYRUA remained stable in magnitude and in spatial distribution pattern. The counties of high–high type were concentrated primarily in the mountainous areas (i.e., Wu Mountains in western Hubei, Xuefeng Mountains in western Hunan). The low–low type was distributed chiefly in the central areas of the Wuhan metropolis, Changsha–Zhuzhou–Xiangtan Metropolis, and Poyang Lake city group, which are surrounding counties of major cities. The distribution of the high–low type was apparent in the counties surrounding Nanchang, there were few low–high types distributed discretely during the study period, and the overall LISA clustering pattern of average ESV did not change significantly during the study period (Figure 3).

### 3.2. Spatial Regression

The Moran’s *I* analysis indicates that spatial regression models for spatial estimation and verification should be established. After OLS estimation, a Moran’s *I* index residual test was performed on the residuals; the Moran’s *I* coefficients in 1995, 2005, and 2015 were 0.400, 0.426, and 0.398, respectively. The *p*-value was 0.001, indicating strong spatial autocorrelation among the residuals. The spatial dependence diagnostics by the OLS model further showed that statistically significant spatial lag and spatial error terms existed in the model residuals (Table 5). The OLS estimation in the classical linear regression model may have errors in model design with no consideration of spatial autocorrelation. Based on the measures of model fit to the data—AIC, SC, and log-likelihood (Table 5 and Table 6)—the performance of the spatial regression models was better fitted to the data than that of the OLS model. Among the three spatial regression models, the SEMLD had the best fit to the data, with the lowest AIC and SC values and highest log-likelihood.

The regression results of the SEMLD are shown in Table 6. It was unanticipated that population density was insignificant in all the models from 1995 to 2015. The possible reason is that other determinants, such as the proportion of developed land, have advantages over population density, and the impacts of population density were not obvious during these periods. Similar results can be found in Hu et al. (2015) [28]. There was a negative spatial association between road density and ecosystem services, but not in all models. Different levels of road density had different impacts on ecosystem services. Similar results can be found in other studies—i.e., that an increase in the density of roads is associated with a decrease in ecosystem services by leading to landscape fragmentation and strengthening socioeconomic activities [52,53].

Distance to a socioeconomic center proved to be an important spatial determinant for ecosystem services, and there was a positive relationship between ecosystem services and distance to a socioeconomic center. Elevation exhibited a significant positive association with ecosystem services in the years studied, indicating that counties at higher elevation tend to have higher ecosystem services. An increase of 100 m in elevation led to an increase of average ESV by 0.200, 0.136, and 0.148 in SEMLD in 1995, 2005, and 2015, respectively. Precipitation proved statistically significant only in OLS models in 1995 and 2015; the positive relationship suggests that the increase in precipitation promoted the increase of ecosystem services. 

River density and ecosystem services were negatively associated in the OLS and SLM models. Numerous studies have provided important evidence that settlements tend to be formed closer to water bodies or rivers [54,55], which inevitably damages the ecosystem. In an additional Pearson’s correlation analysis of river density and construction land, a significant negative correlation was observed in the MRYRUA from 1995 to 2015 (r = 0.111 and *p* = 0.05 in 1995, r = 0.125 and *p* = 0.05 in 2005, and r = 0.115 and *p* = 0.05 in 2015). The proportion of developed land was negatively associated with ecosystem services in all models in 1995, 2005, and 2015. A 1% increase in the proportion of developed land contributed to decreases of 0.497%, 0.402%, and 0.566% in ecosystem services in SEMLD in 1995, 2005, and 2015, respectively. The negative association between the proportion of developed land and ecosystem services indicates that counties with a higher proportion of developed land tended to have lower ecosystem services. An increase in the proportion of developed land means an increase in the consumption of ecosystem services related to human activities, and the literature has shown that an increase in the proportion of developed land leads to ecosystem deterioration [28,56,57]. A statistically significant positive association can be observed between the percentage of forestland and ecosystem services. Using SEMLD for interpretation, 1% of the percentage of forestland increase corresponds to a 0.410%, 0.340%, and 0.319% increase of average ESV, in 1995, 2005, and 2015, respectively. 

Spatial lag terms in the SEMLD were statistically significant in 1995, 2005, and 2015, indicating that the spatial spillover effect of ecosystem services was common in the MRYRUA. The ecosystem services in one county were not only closely related to the individual elements (physical and social factors) but also to other neighborhood factors (ecosystem services in adjacent counties). Each 1% increase in average ESV in a neighboring county correlated to 0.276%, 0.134%, and 0.121% increases in each county in 1995, 2005, and 2015, respectively. Moreover, the spatial error terms in the SEMLD were statistically significant in 1995, 2005, and 2015, documenting that ecosystem services were impacted not only by the driving forces studied in this study but were also influenced by other, omitted variables.

## 4. Discussion and Implications

### 4.1. A Summary of the Findings

National strategies like the Central China Rising Strategy, the Yangtze River Economic Belt Strategy, and the Triangle of Central China Strategy have greatly promoted urbanization and industrialization in the MRYRUA. As a consequence, a considerable amount of farmland has been converted into construction land. In response, a series of land-use policies, such as the Basic Farmland Protection Regulation, the Farmland Balance Policy, and the increasing versus decreasing balance policy, have been implemented in an attempt to control urban expansion and prevent the decline of farmland [58,59]. However, because of the huge economic benefits gap between farmland and construction land, the targets for construction land expansion control and farmland protection were not met [60]. Additionally, land-use activities, such as deforestation and cultivation, as well as forestry engineering in the shelterbelt program in the upper and middle reaches of the Yangtze River basin, resulted in frequent conversion between farmland and forestland.

Spatial determinants identified in this study further showed negative associations among ecosystem services and road density, the proportion of developed land, and river density, while the distance to a socioeconomic center, proportion of forestland land, elevation, and precipitation were positively associated with ecosystem services. Previous studies have evidenced that developed land tends to expand along road networks [38]. The road network affects the flow of elements and resources within and among regions, and the circulation and accumulation of labor, capital, technology factors, and resources affect population redistribution and land development, directly or indirectly. The geographic characteristic (such as distance to a socioeconomic center) has an evident impact on land-use change [61]. The availability and accessibility of multiple resources in urban centers exacerbate the agglomeration of socioeconomic activities. Ecosystem services around the central counties of cities and surrounding counties are becoming increasingly important. Previous studies provide empirical evidence that human settlements are more likely distributed near water bodies because of invaluable natural amenities [55]. River density is therefore negatively associated with ecosystem services. The positive association between the proportion of forestland and ecosystem services shows that an increase in forestland promotes an increase in ecosystem services. Reforestation is an effective means for improving ecosystem services [28]. The ecosystem services in the MRYRUA display significant spatial autocorrelation and spillover effects, indicating that the ecological conservation and land-use policies in a single area cannot completely solve the problem of regional ecological issues and land-use problems. The prevention and control of ecological issues needs effective coordination across counties, especially in urban agglomerations.

### 4.2. Policy Implications

In practice, there always exist conflicts among various plans (e.g., economic and social development planning, environmental protection planning, and land-use planning). For example, land-use planning tends to ignore ecological impacts. In response, multiple planning integration efforts have been promoted in China to balance the differences among various spatial plans and avoid unfavorable outcomes. For example, the three designation zones (agricultural, ecological, and urban) and three lines (urban development boundary, ecological red line, and permanent basic farmland red line) are to alleviate the issues of the decline of farmland and the deterioration of ecosystem services due to the expansion of construction land [62]. Therefore, it is necessary to strategically optimize national spatial planning and ecosystem services management. Empirical research shows that the evolution of regional ecosystem services is impacted not only by physical and human factors but also by the charateristics of the neighboring areas. It is noteworthy that ecosystem services in the MRYRUA exhibit significant spatial autocorrelation and spatial spillover features. In other words, regional ecosystem services are affected by ecological regulation and industrial structure as well as by neighboring counties and more distant counties; the ecological conservation regulation policy for a single area cannot completely solve the problem of regional ecological issues [10]. The effective control of ecological issues requires intercounty joint efforts.

### 4.3. Limitations and Future Research Directions

The revised benefit transfer method and an integrated spatial regression were used to measure the spatial features and the influence mechanism of ecosystem services. However, this study focused only on the supply capacity of the ecosystem services; their demand capacity was an essential aspect of ecosystem services that also should be considered [9]. The study examined only the impact mechanism of total ESV; future research could pay attention to the driving forces of regulating services, supplying services, supporting services, and cultural services. Furthermore, an integrated cross-sectional spatial regression approach was used to identify the spatial determinants of ecosystem services; dynamic spatial panel data models that blend the inter-individual differences and intra-individual dynamics could be considered in future research [63].

## 5. Conclusions

In this study, we examined the spatiotemporal pattern of the ESV in the MRYRUA from 1995 to 2015 using a revised benefit transfer method. Then we identified the spatial autocorrelation of ESV with the global Moran’s *I* index and local Moran’s *I* index. We ultimately analyzed the spatial determinants of average ESV using an integrated spatial regression approach and found a gradually increasing trend in ESV. Forestland provided the greatest proportion of ESV (more than 75%) in the MRYRUA, and farmland and water areas provided more than 10% of the total ESV. The hydrological regulation function accounted for the largest proportion of the total ESV (more than 15%) among all the ecosystem functions. We observed evident spatial dependence and spatial spillover effects of average ESV in the MRYRUA from 1995 to 2015. The average ESV in an area was impacted not only by its factors but also by the characteristics of its neighboring areas due to the spatial spillover effects. The spatial regression results showed that road density, river density, and proportion of developed land were negatively associated with the average ESV, while elevation and precipitation were positively associated with the average ESV. Distance to a socioeconomic center was also found to be an important spatial determinant for average ESV. The results in this study provided important implications for the cross-regional collaborative management of ecosystem services and sustainable land use.

## Figures and Tables

**Table 1:** Area and changes of land-use types in the Middle Reaches of the Yangtze River Urban Agglomerations (MRYRUA) from 1995 to 2015.

Land Use Type	Units	1995	2005	2015	1995–2005	2005–2015	1995–2015
Farmland	Area (km^2^)	176,032.81	174,649.62	170,191.07	−1383.19	−4458.55	−5841.74
	Proportion (%)	31.17	30.93	30.14	−0.24	−0.79	−1.03
Forestland	Area (km^2^)	330,189.84	331,286.12	327,894.71	1096.28	−3391.41	−2295.13
	Proportion (%)	58.47	58.67	58.07	0.19	−0.60	−0.41
Grassland	Area (km^2^)	21,920.51	20,241.30	20,585.03	−1679.20	343.73	−1335.47
	Proportion (%)	3.88	3.58	3.65	−0.30	0.06	−0.24
Water area	Area (km^2^)	25,857.54	27,360.30	28,500.41	1502.76	1140.11	2642.87
	Proportion (%)	4.58	4.84	5.05	0.26	0.21	0.47
Construction land	Area (km^2^)	10,575.69	11,055.84	17,402.33	480.15	6346.48	6826.64
	Proportion (%)	1.87	1.96	3.08	0.09	1.12	1.21
Unused land	Area (km^2^)	95.3	78.52	97.66	−16.78	19.14	2.35
	Proportion (%)	0.02	0.01	0.02	0	0	0

**Table 2:** Transition matrix of land use from 1995 to 2015 in the MRYRUA.

Year	Land Use Type	Farmland	Forestland	Grassland	Water Area	Construction Land	Unused Land
1995–2015	Farmland	**161,659.56**	5906.48	538.05	1299.92	781.48	5.58
	Forestland	5459.57	**320,176.40**	1814.89	297.64	137.81	8.39
	Grassland	340.46	922.65	**19,248.44**	57.82	13.96	1.70
	Water area	3567.13	786.77	115.73	**23,919.71**	109.77	1.30
	Construction land	5001.00	2375.86	202.15	280.69	**9532.21**	10.41
	Unused land	5.04	21.27	1.22	1.76	0.45	**67.93**
1995–2005	Farmland	**163,167.32**	7641.20	657.95	1596.11	1576.59	10.45
	Forestland	7628.76	**320,480.64**	2467.68	465.45	233.64	9.95
	Grassland	415.38	905.03	**18,681.15**	221.52	16.40	1.83
	Water area	3092.75	641.96	79.69	**23,421.04**	124.19	0.67
	Construction land	1724.81	517.59	33.58	152.23	**8624.30**	3.33
	Unused land	3.79	3.42	0.47	1.19	0.56	**69.08**
2005–2015	Farmland	**159,171.53**	7739.92	491.30	1827.54	956.79	4.17
	Forestland	7279.17	**318,331.52**	1509.28	531.42	238.84	5.62
	Grassland	531.49	2160.83	**17,828.35**	44.50	19.24	1.20
	Water area	2631.06	850.68	232.88	**24,640.65**	144.49	0.69
	Construction land	5026.10	2181.54	178.46	314.46	**9694.74**	7.05
	Unused land	10.42	22.30	1.65	1.75	1.74	**59.80**

Notes: Rows show land-use types in 2015, and columns show land-use types in 1995. The number 5906.48 indicates 5906.48 km^2^ of forestland converted to farmland, while the number 5459.57 indicates 5459.57 km^2^ of farmland converted to forestland during 1995–2015; the other numbers follow the same rule.

**Table 3:** Ecosystem services value (ESVs) of different land-use types from 1995 to 2015 (million US$).

Year	Land Use Type	Supplying Services		Regulating Services				Supporting Services		Cultural Services	
		Food Production	Raw Material	Gas Regulation	Climate Regulation	Hydrological Regulation	Waste Treatment	Soil Formation and Retention	Biodiversity Protection	Recreation and Culture	Total
1995	Farmland	6218.56	2425.24	4477.36	6032.00	4788.29	8643.80	9141.28	6342.93	1057.16	49,126.63
	Forestland	3908.02	35,290.64	51,159.59	48,198.96	48,435.81	20,369.09	47,606.84	53,409.66	24,632.39	333,011.01
	Grassland	336.79	281.97	1174.87	1221.86	1190.53	1033.88	1754.47	1464.67	681.42	9140.46
	Water area	398.88	264.43	1308.69	6996.09	14,435.87	13,109.26	1075.63	3191.04	4091.88	44,871.76
	Unused land	0.06	0.13	0.19	0.41	0.22	0.83	0.54	1.27	0.76	4.43
2005	Farmland	6238.38	2432.97	4491.63	6051.23	4803.55	8671.34	9170.41	6363.14	1060.52	49,283.17
	Forestland	3935.44	35,538.25	51,518.54	48,537.14	48,775.65	20,512.01	47,940.86	53,784.40	24,805.22	335,347.51
	Grassland	313.19	262.20	1092.52	1136.22	1107.08	961.42	1631.49	1362.01	633.66	8499.79
	Water area	429.32	284.61	1408.56	7530.02	15,537.60	14,109.74	1157.72	3434.58	4404.17	48,296.33
	Unused land	0.05	0.11	0.16	0.36	0.19	0.71	0.47	1.09	0.66	3.80
2015	Farmland	6083.02	2372.38	4379.78	5900.53	4683.93	8455.40	8942.04	6204.68	1034.11	48,055.87
	Forestland	3965.86	35,812.95	51,916.76	48,912.32	49,152.67	20,670.56	48,311.43	54,200.13	24,996.96	337,939.63
	Grassland	326.22	273.11	1137.96	1183.48	1153.13	1001.41	1699.36	1418.66	660.02	8853.34
	Water area	443.93	294.29	1456.49	7786.24	16,066.30	14,589.85	1197.12	3551.45	4554.03	49,939.70
	Unused land	19.82	7.73	14.27	19.22	15.26	27.54	29.13	20.21	3.37	156.54
1995–2005	Farmland	27.42	247.61	358.95	338.18	339.84	142.92	334.02	374.74	172.83	2336.51
	Forestland	−23.61	−19.76	−82.35	−85.64	−83.45	−72.47	−122.97	−102.66	−47.76	−640.67
	Grassland	30.44	20.18	99.88	533.93	1101.73	1000.48	82.09	243.54	312.29	3424.56
	Water area	−0.01	−0.02	−0.03	−0.06	−0.03	−0.12	−0.08	−0.18	−0.11	−0.63
	Unused land	−155.36	−60.59	−111.86	−150.69	−119.62	−215.94	−228.37	−158.46	−26.41	−1227.31
2005–2015	Farmland	30.42	274.70	398.22	375.18	377.02	158.55	370.57	415.73	191.74	2592.12
	Forestland	13.03	10.91	45.44	47.26	46.05	39.99	67.86	56.65	26.36	353.55
	Grassland	14.61	9.68	47.93	256.22	528.70	480.11	39.39	116.87	149.86	1643.37
	Water area	0.01	0.03	0.04	0.09	0.05	0.18	0.12	0.27	0.16	0.95
	Unused land	−135.54	−52.86	−97.59	−131.47	−104.37	−188.40	−199.24	−138.25	−23.04	−1070.76
1995–2015	Farmland	57.84	522.31	757.17	713.35	716.86	301.47	704.59	790.47	364.56	4928.63
	Forestland	−10.58	−8.86	−36.91	−38.38	−37.40	−32.48	−55.11	−46.01	−21.40	−287.12
	Grassland	45.05	29.86	147.81	790.16	1630.43	1480.59	121.48	360.40	462.15	5067.94
	Water area	0.00	0.01	0.01	0.03	0.02	0.06	0.04	0.09	0.06	0.33
	Unused land	19.82	7.73	14.27	19.22	15.26	27.54	29.13	20.21	3.37	156.54

**Table 4:** Variable descriptions and data sources.

Variable Category	Variable	Description	Data Sources
Dependent variable	AESV	Average ecosystem services value	Calculated from Section 2.2.2
Physical driving forces	Elevation (m)	Average elevation	Geospatial Data Cloud Site, Computer Network Information Center, Chinese Academy of Sciences (http://www.gscloud.cn)
	Precipitation (mm)	Annual average precipitation	Data Center for Resources and Environmental Sciences, Chinese Academy of Sciences (RESDC) (http://www.resdc.cn)
	River density (km/km^2^)	River length per square kilometer	National Geomatics Center of China (NGCC) (http://ngcc.sbsm.gov.cn/)
	Proportion of developed land	Total developed land divided by the administrative area	Extracted from LULCC data
	Proportion of forestland land	Total forestland divided by the administrative area	Extracted from LULCC data
Socioeconomic driving forces	Population density (person/km^2^)	Total population divided by the administrative area	Data Center for Resources and Environmental Sciences, Chinese Academy of Sciences (RESDC) (http://www.resdc.cn)
	Railway density (km/km^2^)	Railway length per square kilometer	National Geomatics Center of China (NGCC) (http://ngcc.sbsm.gov.cn/)
	Highway density (km/km^2^)	Highway length per square kilometer	National Geomatics Center of China (NGCC) (http://ngcc.sbsm.gov.cn/)
	National road density (km/km^2^)	National road length per square kilometer	National Geomatics Center of China (NGCC) (http://ngcc.sbsm.gov.cn/)
	Distance to socioeconomic center (km)	Distance to socioeconomic center	Calculated by ArcGIS10.3 software’s Near tool

**Table 5:** Regression results of the ordinary least squares (OLS), spatial lag model (SLM), and spatial error model (SEM) from 1995 to 2015.

Variable	1995			2005			2015		
	OLS	SLM	SEM	OLS	SLM	SEM	OLS	SLM	SEM
Population density	−0.104 (0.094)	−0.086 (0.086)	−0.085 (0.073)	−0.009 (0.098)	0.008 (0.086)	0.020 (0.075)	−0.050 (0.088)	−0.033 (0.080)	−0.001 (0.071)
Railway density	−0.063 (0.055)	−0.051 (0.051)	−0.004 (0.042)	−0.174 * (0.093)	−0.180 ** (0.082)	−0.161 ** (0.066)	−0.012 (0.096)	−0.049 (0.089)	−0.017 (0.067)
Highway density	0.006 (0.046)	0.025 (0.042)	−0.030 (0.041)	−0.021 (0.048)	−0.009 (0.042)	−0.006 (0.041)	−0.078 * (0.041)	−0.087 ** (0.038)	−0.056 (0.035)
National road density	−0.076 (0.052)	−0.088 * (0.048)	−0.022 (0.037)	−0.107 ** (0.054)	−0.126 *** (0.048)	−0.049 (0.038)	−0.072 (0.058)	−0.097 * (0.054)	−0.039 (0.043)
Distance to socioeconomic center	0.061 ** (0.030)	0.055 ** (0.028)	0.030 (0.035)	0.086 *** (0.031)	0.075 *** (0.028)	0.096 *** (0.035)	0.042 (0.029)	0.043 * (0.026)	0.075 ** (0.033)
Proportion of developed land	−0.383 *** (0.073)	−0.284 *** (0.069)	−0.437 *** (0.057)	−0.367 *** (0.077)	−0.226 *** (0.071)	−0.367 *** (0.061)	−0.558 *** (0.085)	−0.402 *** (0.082)	−0.527 *** (0.067)
Proportion of forestland land	0.238 *** (0.027)	0.185 *** (0.027)	0.394 *** (0.031)	0.216 *** (0.027)	0.164 *** (0.026)	0.326 *** (0.031)	0.193 *** (0.026)	0.158 *** (0.025)	0.308 *** (0.030)
Elevation	0.124 ** (0.048)	0.059 (0.045)	0.179 *** (0.059)	0.150 *** (0.048)	0.055 (0.044)	0.124 ** (0.058)	0.201 *** (0.044)	0.105 ** (0.043)	0.135 ** (0.055)
Precipitation	0.053 ** (0.026)	0.026 (0.024)	0.113 (0.076)	0.004 (0.025)	−0.011 (0.022)	0.020 (0.057)	0.041 * (0.024)	0.016 (0.022)	0.054 (0.053)
River density	−0.132 *** (0.040)	−0.133 *** (0.037)	−0.013 (0.028)	−0.089 *** (0.040)	−0.092 ** (0.035)	−0.001 (0.029)	−0.092 ** (0.037)	−0.095 *** (0.034)	−0.005 (0.028)
Spatial lag term		0.359 *** (0.058)			0.419 *** (0.054)			0.334 *** (0.053)	
Spatial error term			0.827 *** (0.034)			0.753 *** (0.042)			0.742 *** (0.043)
Constant	0.503 *** (0.025)	0.324 *** (0.034)	0.345 *** (0.051)	0.570 *** (0.026)	0.330 *** (0.035)	0.462 *** (0.041)	0.576 *** (0.023)	0.390 *** (0.034)	0.468 *** (0.036)
Moran’s *I* (error)	0.400 ***			0.426 ***			0.398 ***		
LM (lag)	47.520 ***			67.119 ***			43.151 ***		
Robust LM (lag)	10.205 **			2.860 *			3.965 *		
LM (error)	132.710 ***			150.926 ***			131.176 ***		
Robust LM (error)	95.395 ***			86.667 ***			91.989 ***		
LM (lag and error)	142.915 ***			153.786 ***			135.141 ***		
*Measures of fit*									
Log likelihood	341.339	360.724	415.646	339.456	367.692	409.750	367.818	386.784	431.184
AIC	−660.678	−697.449	−809.292	−656.913	−711.383	−797.501	−713.636	−749.568	−840.368
SC	−619.056	−652.043	−767.670	−615.291	−665.977	−755.878	−672.014	−704.162	−798.746
*N*	325	325	325	325	325	325	325	325	325

Notes: The study uses the queen’s contiguity weight matrix. *** *p* ≤ 0.01, ** *p* ≤ 0.05, * *p* ≤ 0.1. Standard errors are in parentheses. LM = Lagrange multiplier. AIC = Akaike information criterion. SC = Schwarz criterion.

**Table 6:** Regression results of the spatial error models with lag dependence from 1995 to 2015.

Variable	1995	2005	2015
Population density	−0.074 (0.071)	0.021 (0.074)	0.002 (0.070)
Railway density	−0.014 (0.041)	−0.156 ** (0.065)	−0.006 (0.066)
Highway density	−0.041 (0.039)	−0.001 (0.040)	−0.045 (0.035)
National road density	0.001 (0.036)	−0.035 (0.038)	−0.025 (0.043)
Distance to socioeconomic center	0.047 (0.035)	0.107 *** (0.036)	0.086 ** (0.034)
Proportion of developed land	−0.497 *** (0.057)	−0.402 *** (0.063)	−0.566 *** (0.069)
Proportion of forestland land	0.410 *** (0.030)	0.340 *** (0.031)	0.319 *** (0.030)
Elevation	0.200 *** (0.059)	0.136 ** (0.060)	0.148 *** (0.056)
Precipitation	0.096 (0.086)	0.025 (0.063)	0.056 (0.057)
River density	0.003 (0.027)	0.008 (0.029)	0.002 (0.027)
Spatial lag term	−0.276 *** (0.071)	−0.134 * (0.071)	−0.121 * (0.066)
Spatial error term	0.864 *** (0.029)	0.790 *** (0.038)	0.774 *** (0.040)
Constant	0.499 *** (0.073)	0.533 *** (0.062)	0.533 *** (0.056)
*Measures of fit*			
Log likelihood	422.660	411.177	432.621
AIC	−821.320	−798.355	−841.242
SC	−775.914	−752.949	−795.836
*N*	325	325	325

Notes: The study uses the queen’s contiguity weight matrix. *** *p* ≤ 0.01, ** *p* ≤ 0.05, * *p* ≤ 0.1. Standard errors are in parentheses. LM = Lagrange multiplier. AIC = Akaike information criterion. SC = Schwarz criterion.
